# The Graphic Descriptor Ontology: An ontology for semantic annotation of graphics and its application to graphic libraries of anatomical standards

**DOI:** 10.1371/journal.pone.0347931

**Published:** 2026-04-29

**Authors:** Melissa D. Clarkson, Noah C. Perry, Landon T. Detwiler

**Affiliations:** 1 Division of Biomedical Informatics, University of Kentucky, Lexington, Kentucky, United States of America; 2 Institute for Biomedical Informatics, University of Kentucky, Lexington, Kentucky, United States of America; Old Dominion University, UNITED STATES OF AMERICA

## Abstract

The information held by visual representations is typically opaque to information processing systems and able to be interpreted only by human viewers. We introduce the Graphics Descriptor Ontology (GDO) to serve as an ontology for domain-independent annotation and description of graphics and their elements. Our goal is to represent information about graphics that corresponds to what a human observer could conclude from viewing a graphic or that would help to inform a viewer about a graphic. This work builds upon ontological modeling of information content entities and uses theories and vocabularies from the fields of semiotics, visual arts, technical communication, and computer graphics. We define a graphic as a spatial composition composed of graphical marks. The three types of graphical marks are line mark, point mark, and region mark. We present an approach to representing roles and qualities for information content entities, including graphical marks and graphics. Anatomical graphics serve as our use cases, and we provide an anatomy extension for the GDO to model anatomy-specific content. We introduce our work as an illustrated ontology available through a web browser, accompanied by over 100 explanatory graphics.

## 1. Introduction

Visual representations are necessary for human communication and reasoning in domains such as the life sciences and medicine. Information systems have traditionally relied on textual representations for processing and storing information, with visuals serving as supplements to this information rather than as bearers of computable information themselves. Tags or captions may be associated with visual representations and available for search and retrieval in systems, but interpretation of visual information is traditionally limited to humans, rather than computers.

In this report we describe an ontology for use in annotation of graphics. The purpose is to provide machine-readable information about graphics for use by semantically-aware information systems. Our goal is to represent information that a human observer could conclude from viewing a graphic or that would help to inform a viewer about a graphic. We introduce the Graphics Descriptor Ontology (GDO) to serve as an ontology for domain-independent annotation of graphics and their elements. Anatomical graphics serve as our use cases, and we provide the anatomy extension of the GDO to model anatomy-specific content. We refer to the combined GDO and the anatomy extension as the GDO/A.

We create libraries of anatomical graphics intended to serve as visual standards. They represent types of anatomy, rather than the anatomy of a specific individual, by depicting morphology and features typical of anatomical structures, phenotypes, and malformations. For example, our graphic library depicting phenotypes of cleft lip presents a series of unilateral and bilateral clefts in which only essential characteristics relevant to classifying phenotypes differ among graphics (the extent of the cleft(s) and deformation of the ala of the nose). The graphics address what has been called “the linguistic problem of morphology” [1], which arises from the difficulty in using terms and terminologies to precisely communicate about biological forms and their characteristics. The GDO/A is designed to be broadly applied and can be used to describe graphics that depict types of anatomy as well as graphics that show the anatomy of a particular individual. We envision that semantically-augmented graphics of anatomy will be useful in designing intelligent information systems to assist in healthcare, medical education, and biocuration.

### 1.1. Related work in visual representation

This project draws upon work from multiple fields including semiotics, visual arts, technical communication, and computer graphics. This section highlights some of the work in these disciplines that influenced development of the GDO.

#### 1.1.1. The nature of representations and their meanings.

**Fig 1:**
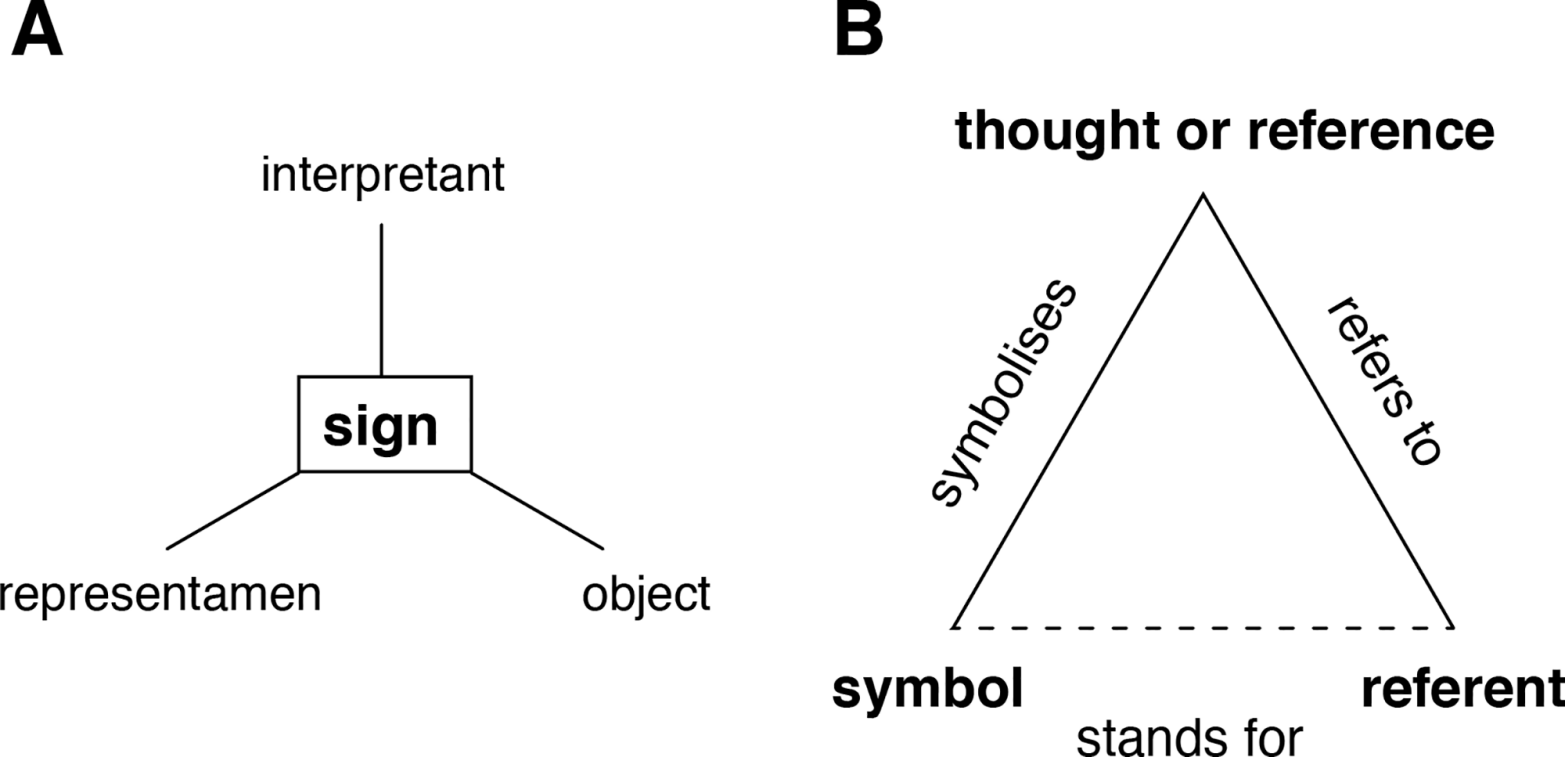
Two models of semiotics. **(A)** Pierce’s three-part model of a sign. **(B)** Ogden and Richard’s model that makes explicit the relations between the three factors necessary for a sign to have meaning.

Semiotics is the study of signs and sign systems. Its origins lie in the study of language, but signs also include images and gestures. Signs have been a subject of study since the time of Aristotle, with numerous philosophers proposing theories to account for how signs hold meaning [2,3]. Philosopher and logician Charles Sanders Peirce offers a three-part model for signs, in which a sign is the union of (1) a *representamen* (or “sign vehicle”), which stands for something to someone and is perceptible; (2) an *object*, the material object, process, or idea for which the representamen stands; and (3) an *interpretant*, which is created in the mind of the one perceiving the representamen and described by Peirce as a “mediating representation” [4]. The meaning of a sign arises from its interpretation by the interpreter. Later authors have represented Pierce’s model using a tripod-shape diagram similar to that in Fig 1A [3].

Subsequent work by Charles Kay Ogden and Ivor Armstrong Richards uses a similar three-part model, but emphasizes relations among the three components [5,6]. As shown in Fig 1B, a *thought or reference* forms in one’s mind as one perceives or thinks about a *referent*. The thought or reference is said to *refer to* the referent. A *symbol* is defined as “those signs which men use to communicate one with another and as instruments of thought.” A symbol *symbolizes*, or evokes, a thought or reference when it is perceived [6]. These two relations—a thought referring to a referent and a symbol symbolizing a thought—are causal relations. The relation between a symbol and its referent is of a different type. Ogden and Richard characterize it as an imputed (or indirect) relation “which consists in [the symbol] being used by someone to stand for a referent”.

#### 1.1.2. Practices in the visual arts.

When using traditional media, artists and illustrators render *marks* on a substrate (material plane) from which pictures are constructed. These marks include pencil lines on paper, strokes of oil paint on canvas, and lines scratched into a metal etching plate to be printed as an engraving. As postulated in art theory, the fundamental units of drawings are *points* (the most elemental mark), *lines* (the perceivable paths of points), and *planes* (surfaces that extend in height and width, formed by lines closing upon themselves or the joining of multiple lines) [7,8].

The expressive qualities of line marks have applications in technical and scientific communication. For example, use of different line weights (thicknesses) can reduce ambiguity in technical illustrations [9] and the expressivity of lines may have applications in interpreting data visualizations [10]. In the field of natural science and medical illustration, variations in the curvature or placement of lines may communicate key characteristics for classifying biological specimens or a patient’s phenotype.

#### 1.1.3. Conventions in technical drawings.

Architecture, product design, and engineering all rely on technical drawings to communicate the specifications of objects that haven been, or will be, constructed. Blueprints and product sketches use lines to represent many different types of things, including the contours of an object, edges of planes formed by cutting through an object, hidden edges of an object, and arrows that point to areas of an object [11,12]. The vocabulary of lines in blueprints is represented using different line weights and styling (solid, dotted, dashed).

*Projections* are techniques for representing a three-dimensional object as a two-dimensional graphic. They are based on an imaginary scenes consisting of the viewer and the referent object, with “lines of sight” extending between the viewer and the object. Projection techniques differ in how the lines of sight intersect with the projection plane (also known as the view plane or image plane) that is represented by the drawing. Blueprints use *parallel projections* in which the lines of sight are parallel. In contrast, artists and illustrators typically use *perspective projections* in which the lines of sight converge at a point in space known as the center of projection after passing through the projection plane. This creates the illusion of depth.

#### 1.1.4. Line drawings in computer science.

Research on algorithmic methods to produce line drawings has the goal of mimicking the process by which artists select lines to represent an object’s form. This work has led to improved characterization of line types and mathematically-based definitions. Occluding contours, those lines drawn at the boundary of an object to create a silhouette, are easily reproduced. But lines used by artists to represent valleys, ridges, and suggestive contours are more challenging to identify from the topology of an object [13–17].

To assist in creating the appearance of three-dimensional form in line drawings, artists may augment drawings with hatching (lines that follow contours) or stippling (fields of dots) to simulate tone (shades of gray). Methods for algorithmically simulating each of these techniques have been investigated [18–20].

#### 1.1.5. Types of visual representations.

A photograph of a landscape, a painted portrait, and a diagram of a wiring scheme are all visual representations that differ not only in the subject represented, but in the type of representation.

Extending his three-part model of signs, Pierce offers a three-category classification for types of signs. *Indexical signs* have a direct physical or causal connection between the representamen (sign-vehicle) and the referent. Examples include natural signs, such as smoke standing for fire; medical symptoms, such as a rash standing for contact with poison ivy; and visual or audio recordings, such as a photograph standing for the person or thing that was photographed. *Symbolic signs* have an arbitrary relationship between the representamen and referent. These signs must be agreed upon and learned. They include words, numbers, symbols such as “+”, and national flags. *Iconic signs* have a perceived resemblance between the representamen and the referent. Portraits, schematic diagrams, and replicas such as model trains are iconic signs in Pierce’s typology [3].

### 1.2. Related work in ontological representation

#### 1.2.1. Ontological approaches to representing information entities.

Several ontologies have been developed to represent information entities. They take slightly different approaches to distinguishing between the informational content of the entities and the artifacts that bear the information (reviewed in [21]).

The Basic Formal Ontology (BFO) is an upper-level ontology that has been widely adopted for building ontologies to describe domains within biomedical informatics [22–24]. The BFO classifies all entities as either a *continuant* (an entity that persists over time) or an *occurrent* (an entity such as an event or process that unfolds in time.) The Information Artifact Ontology (IAO) extends the BFO to model information content entities, particularly those used in biomedical documentation. The central idea underpinning the IAO is that an *information content entity* is about some other entity and it is dependent upon some material entity to bear (or concretize) the information [25]. For example, the information content of some particular magazine article is about a person and is dependent upon the ink and paper it is printed on. In this model, information can be thought of as a pattern (such as a sequence of letters) designed by a creator to represent something. The pattern depends upon some material entity (the ink and paper) for its existence. The pattern can be copied to some other material entity that will serve as another bearer of that information. An information content entity is classified as a *generically dependent continuant* because information can migrate from one bearer to another. This contrasts with a *specifically dependent continuant* that depends upon one or more independent continuants for its existence (for example, the mass of some particular apple and the color of that apple cannot exist without that apple).

The Information Entity Ontology (IEO) is a member of the Common Core Ontologies and also uses the BFO as an upper-level ontology [26]. It models information using an approach similar to IAO. It extends the modeling of information content entities by classifying them as representational, descriptive, designative, and directive.

Both the IAO and IEO model information as information content entities which are characterized as being about some other entity. A different approach is to model information as a role of a representation [27]. In this schema, representations are composed of *form* (what is displayed for human perception, for example a shape of a sculpture or a pattern of letters) and *content* (what is expressed through the representation, such as the thing depicted or the meaning of text). Form encodes content. Information is a role played by a representation in the context of informing.

#### 1.2.2. Domain-specific ontologies for visual representation.

A small number of modeling efforts have addressed the needs of practitioners working with domain-specific pictorial content. Ontologies have been designed for use by art historians and curators of cultural heritage objects to describe the subjects, themes, and interpretations of artwork [28,29] and for geologists to describe sedimentary structures and interpret them for purposes such as petroleum exploration [30,31]. But formal ontologies are not commonly used for describing or categorizing visual representations.

### 1.3. Developing an ontological framework for visual representations

Visual representations have different types of communication purposes and are used in fields ranging from art and advertising to technical communication. The focus of our work is visual representations that communicate information relevant to science and medicine. This narrows the focus of our modeling to attributes relevant to the creation of visual representations and how they correspond to referents. These include:

- *Fundamental unit:* The fundamental units of information of a representation are determined by the method used to create the representation. The information of photographs and clinical radiographs is the set of measurements from a sensor that have been projected onto a two-dimensional plane as values of color and/or luminosity. A pixel is the fundamental unit, with the set of pixels interpreted by the viewer as a picture of the referent. In contrast, the information of a drawing or painting is carried in the spatial composition of its fundamental units (point, line, and region marks) that are interpreted by the viewer as a picture of the referent.
- *Referent type:* Visual representations represent different types of referents. Information may describe objects in the world, processes that take place in the world, or ideas held only in the mind.
- *Information content type:* The information content communicated through a visual representation can be of several types. Information that is a set of measurements would be plotted as a data visualization. If the information is about the relationships among entities, this could be communicated using a representation such as a network diagram, Venn diagram, or wiring diagram. The shape of an object is depicted by a photograph or drawing.
- *Specificity:* Drawings of objects can be characterized as representing either a particular entity in the world (a sketch of a specific archeological specimen) or a general type of entity (an illustration of a typical healthy human heart or typical diseased human heart).

In this paper we introduce the GDO, which is designed to model spatial compositions that serve as visual representations. It distinguishes representations that originate from sensors from those drawn as compositions of points, lines, and regions (which we term “graphics”). The use cases for this work are graphics of anatomical structures. But this project is intended to serve as a foundation for modeling any type of representational information content entity that is a spatial composition, particularly those composed of points, lines, and regions.

## 2. Methods

In this paper we describe both the GDO and the anatomy extension of the GDO, which was developed for annotating our anatomical graphics. This modular construction allows the GDO to be extended to model other types of graphics by developing other domain-specific extensions.

### 2.1. Content development

The GDO/A was authored using the Web Ontology Language (OWL) [32] and Protégé desktop [33]. The GDO is rooted in the Basic Formal Ontology (BFO, version 2019-08-26) and uses several classes from the Information Entity Ontology (IEO) of the Common Core Ontologies (version 2024-11-06) as a mid-level ontology. Because only a small number of classes from the BFO and IEO are needed to structure our class hierarchy, we include only the needed portions rather than importing the entire ontologies. Development followed the principles of the Open Biological and Biomedical Ontology Foundry, which includes supplying a textual definition for each class and reusing relations from the Relation Ontology [34]. New versions are released as content is added, with version numbers represented in the ontology version IRI.

Classes of the GDO were initially developed by gathering terms and definitions used within the fields of semiotics, visual communication, illustration, and technical drawing. Terms were identified based on literature searches, web searches using Google Search, and one author’s (MDC) training in visual design. Synonyms were identified and hierarchical relationships created. Initial work was supplemented and refined using vocabularies from literature describing algorithmic methods to produce line drawings. The taxonomy of lines offered by [12] was of particular help in creating classes of line mark roles. The vocabulary of Denman Ross was used to name classes for values of graphical marks (the lightness or darkness of a mark) [35].

For the anatomy extension, anatomy-specific roles for region marks (such as “region representing an artery”) were included if deemed relevant to the anatomical graphics our group anticipates developing over the next several years. The classes for roles of region marks were mapped to anatomical classes from Foundational Model of Anatomy (FMA, version 5.0.0) [36] and Uberon (release 2024-09-03) [37]. Mappings are based our experience as members of the FMA development team. Classes for anatomical views provide information about a scene that is depicted by describing the relative position between a referent and the line of sight of the viewer (such as a right lateral view or a posterior view). These were created to describe our anatomical graphics and to correspond to many of the anatomical directions represented in the Biological Spatial Ontology [38].

### 2.2. Use cases

The GDO/A was developed concurrently with the first two graphic libraries we designed to serve as visual standards for anatomy. These libraries depict types of variations in human interparietal bones and phenotypes of cleft lip (both available at https://graphics.endlessforms.info). We employed the GDO/A in our process for semantically augmenting these graphics [39]. This process tested the coverage and specificity of the GDO/A and we expanded modeling as needed.

### 2.3. Development of explanatory graphics and a web browser

The GDO/A is presented as an *illustrated ontology* through a web application that provides visual explanations or examples for selected classes and individuals. The explanatory graphics were annotated with identifiers from the GDO following the same process we use for our anatomical graphics, and therefore served as additional use cases for the GDO.

## 3. The Graphic Descriptor Ontology and the anatomy extension

The BFO was used as the upper-level ontology to align this project with knowledge representation practices in biomedical domains. Several classes from the IEO of the Common Core Ontologies are used as a bridge between the BFO and content developed for the GDO. In the following description, class names are in bold, object properties are in bold and italic, and datatype properties are in italic. IRIs for classes and properties from the IEO are represented using the abbreviated prefix “cco:”, standing for “https://www.commoncoreontologies.org/”. Our discussion makes reference to classes from IAO abbreviated with the prefix “IAO:”, standing for “http://purl.obolibrary.org/obo/”.

### 3.1. Types of information content entities

**Fig 2:**
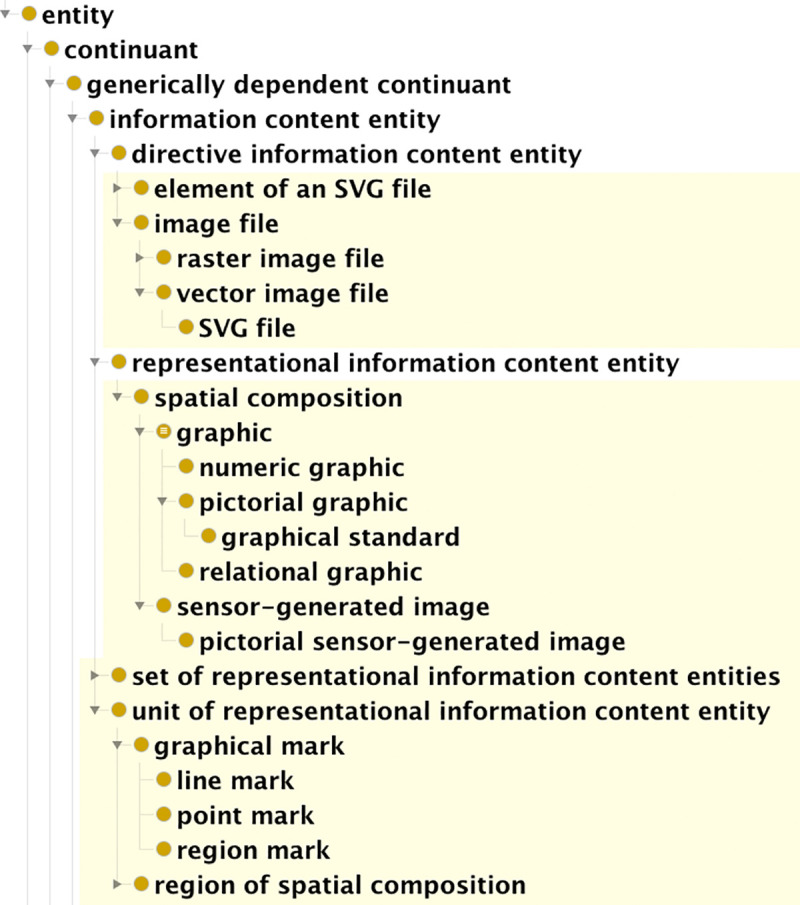
Screen capture of the Protégé interface showing part of the GDO class hierarchy for information content entities. Classes created for the GDO are highlighted in yellow.

To distinguish between information that serves as instructions for rendering graphics and information expressed by the graphics in representing things in the world, we extended classes **directive information content entit**y (cco:ont00000965) and **representational information content entity** (cco:ont00001069) as shown in Fig 2.

Our graphics are distributed as scalable vector graphic (SVG) files. This is an XML-based format that provides instructions for creating an image by drawing a set of shapes with curved or straight lines. It also contains instructions for how to display each shape (for example, the color in which to draw a line, whether a line is to be dashed, whether a shape is to be filled with color). To represent these entities, **directive information content entity** was extended with classes for types of image files (including **SVG file**, **JPG file**) and elements of SVG files that specify pieces of visual output (such as **SVG circle element**, **SVG path element**). Directive information content entities do not need to be computer files: instructions to draw an image by hand — perhaps as a connect-the-dots puzzle — would belong to this class.

The IEO defines **representational information content entity** as “An Information Content Entity that represents some Entity.” The examples provided by the authors are (a) the content of a court transcript represents a courtroom proceeding, (b) the content of a photograph of the Statue of the Liberty represents the State of Liberty, and (c) the content of a video of a sporting event represents the sporting event [40]. We extended this class with the subclass spatial composition.

#### 3.1.1. Spatial compositions.

A common feature of photographs, diagrams, drawings, and paintings is that the information communicated by each depends upon the spatial relationships among their components. If pixels or marks are displaced from one location within the composition and moved to another, the information conveyed by the representation is likely changed. Recall that information content entities are generically dependent continuants, with information held by a pattern of elements. For photographs, diagrams, drawings, and paintings, that pattern depends upon the spatial arrangement of their elements. (This is different than for textual representations, in which the information is held as a pattern that relies only on the sequence of characters or symbols.) We developed the class **spatial composition** to encompass this broad category of representational information content entities, defining it as “A representational information content entity for which the information is expressed by an arrangement of elements in two- or three-dimensional space.” Although our current use of the GDO concerns only two-dimensional representations, this broad definition lends itself to future modeling of three-dimensional representations, including replicas such as model trains and prototypes such as architectural models.

Spatial compositions use spatial attributes, such as position and shape, to express information. We provide two subclasses of spatial composition. A **sensor-generated image** is “a spatial composition created through projection of values onto a two-dimensional plane of a sensor, where the values are measurements of a characteristic of some entity and represented as color and/or luminosity.” This definition is broad enough to include not only photos but also images generated by detection of sound and infrared wavelengths that are outside our focus of pictorial representations. Therefore we introduce the subclass **pictorial sensor-generated image**, defined as “a sensor-generated image that represents the form (shape) and/or visual appearance of some referent entity”, to more specifically categorize images such as photographs, radiographs, and micrographs (photographs taken through a microscope). The fundamental unit of information for all sensor-generated images is the pixel.

The second type of spatial composition is a **graphic**. The defining characteristic of a graphic is that it is composed of graphical marks. We define three types of these marks: A **point mark** is a graphical mark rendered within a two- or three-dimensional coordinate system that evokes recognition of a position (rather than an extent) by the human perceptual system. A **line mark** evokes recognition of a one-dimensional line, and a **region mark** evokes recognition of a two-dimensional surface or three-dimensional volume. These classes correspond to the fundamental types of marks in the visual arts. Graphical marks can be rendered in traditional media (using strokes of pencil or paint applied with a brush), using SVG elements, or as pixels in a raster image. There is not a direct correspondence between a type of SVG element (a directive information content entity) and type of graphical mark (a representational information content entity) in the GDO. A SVG path element could draw a line mark (if rendered with only a stroke) or a region mark (if rendered with a fill). A SVG circle element could draw a point mark, line mark, or region mark.

A **graphical mark** is itself not necessarily a representational information content entity because it will often require the context of a composition to serve as information. (For example, a horizontal line might represent the horizon in a landscape, a tightrope, or the top of a table, but unless that line is placed in the context of a drawing it does not convey this information to the viewer.) The superclass of graphical marks is **unit of representational information content entity**. Our thinking is influenced by a proposal by other authors for modeling of representational units in the IAO [25].

Graphical marks can be composed to create different types of graphics, including drawings, plots of data, and diagrams. We provide subclasses of graphic to distinguish among different types of information content, as listed in Table 1

**Table 1:** Types of graphics.

Class label	Definition	Examples
pictorial graphic	A graphic for which the information content is primarily about the form (shape) and/or visual appearance of one or more referents and for which the spatial properties of the spatial composition correspond in some way to spatial properties of the referent.	• a drawing of a fish • an architectural plan for a house • a map of a state showing locations of hospitals
data graphic	A graphic for which the information content is primarily data values that represent categories, measurements, or rankings that are attributes of one or more referents. Data values are encoded as visual variables such as length, size, position, or color within the composition.	• a bar graph showing the number of students in each grade • a scatterplot of height and weight • a bump chart showing rankings in a sports league
relational graphic	A graphic for which the information content is primarily about relationships among referents.	• a network diagram • a Venn diagram • a wiring diagram

#### 3.1.2. Parts of spatial compositions.

There is often a need to refer to some meaningful set of graphical marks in a graphic or region of pixels in a sensor-generated image. Practitioners of visual arts refer to parts of a visual representation as belonging to the foreground (the subject matter) or the background (not the subject matter). We introduce the classes **background of spatial composition** and **foreground of spatial composition** to align with use in the visual arts. In image processing, a region of interest (ROI) is an area of a sensor-generated image that is selected for further investigation or analysis. We provide the class **region of interest in spatial composition** to account for ROIs identified within raster images as well as ROIs within graphics.

### 3.2. Representing graphic standards

We define a **graphical standard** as “a pictorial graphic that depicts a generalized or idealized form that defines a type of entity or carries information relevant to one or more classification schemes or rating schemes.” In our work developing visual standards for anatomy, each graphical standard refers to a type of anatomical entity by using a specific arrangement of points, lines, and regions. This arrangement does not depend on the scale at which the graphic is drawn, the colors of regions, or the media in which the graphic is rendered. Applications that use the graphical standards may display the same standard at different scales and in different colors, and perhaps at different rotations. Graphics with these types of transformations are members of an equivalence class that can be transformed into one another through scaling, translation, and rotation [41]. However, reflection transformations do not preserve equivalence because the laterality of features may be a defining characteristic of the referent.

A single graphical standard may have different directive information content entities for drawing that standard. We distribute files in the SVG format, but a file in JPG or PNG format would create the same composition, carry the same information, and therefore draw the same pictorial standard.

We develop sets of graphical standards related by anatomical themes, such as depicting different phenotypes or malformations of some part of the body. To refer to these collections in the GDO, we use the class **library of graphical standards**. Our modeling categorizes these libraries as a type of information content entity that is a set of representational information content entities. A library of graphical standards represents a set of entities in the world related by some theme. For example, the graphic library depicting cleft lip represents all instances of lips with a phenotype of clefting, even if a particular child’s phenotype is not an exact match to one of the graphic standards.

### 3.3. Qualities

A quality in the BFO is a specifically dependent continuant that inheres in some independent continuant [22]. Information content entities are generically dependent continuants (rather than independent continuants) and therefore we cannot model qualities as inhering in information content entities and remain consistent with BFO modeling. This presents a modeling dilemma. The IEO of the Common Core Ontologies provides the class **Information Quality Entity** (cco:ont00000314) that is defined as “a Quality that concretizes some Information Content Entity” and is classified as a specifically dependent continuant. This does not align with our need to describe attributes of the information itself, not an individual concretized entity.

**Fig 3:**
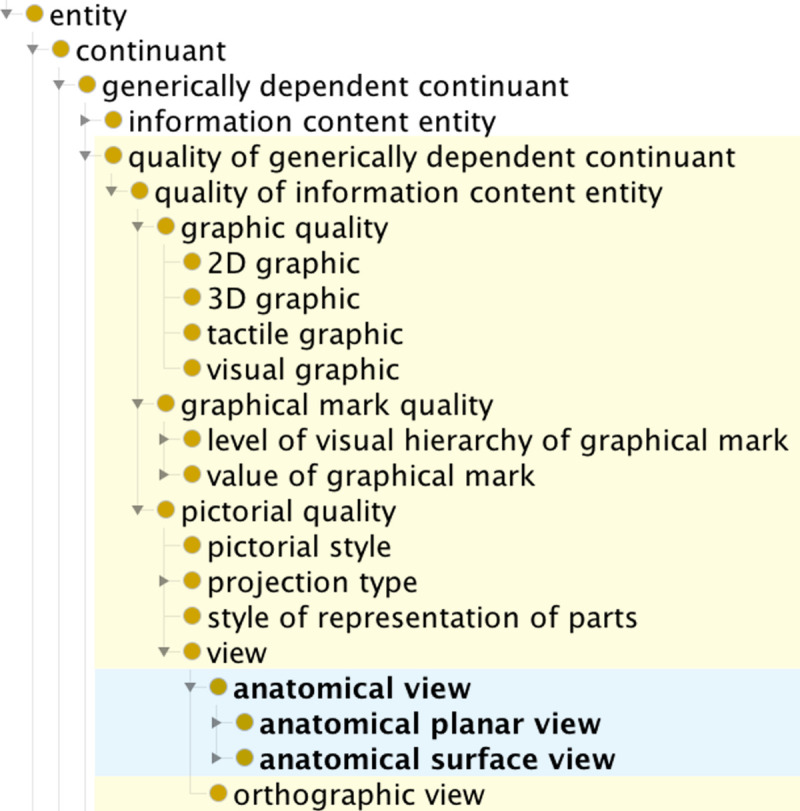
Part of the GDO/A class hierarchy for qualities of generically dependent continuants. Classes created for the GDO are highlighted in yellow. Classes of the anatomy extension are in blue.

To remain consistent with the BFO and extend its hierarchy for our modeling needs, we introduce a new type of generically dependent continuant, **quality of generically dependent continuant**, as shown in Fig 3, defined as “a generically dependent continuant that is an attribute or characteristic of some other generically dependent continuant.”

#### 3.3.1. Qualities of graphics.

A graphic is a representational information content entity consisting of graphical marks. A graphic may have the quality **visual graphic,** meaning that the graphics marks are designed as differences in color or luminosity to be perceived using sight, or **tactile graphic,** in which the graphical marks are designed as raised surfaces to be perceived using touch. **2D quality of graphic** is a quality that specifies the graphical marks are positioned using a two-dimensional (planar) coordinate system. **3D quality of graphic** means the graphical marks are positioned using a three-dimensional (volumetric) coordinate system but projected onto a two-dimensional plane for display.

#### 3.3.2. Qualities of graphical marks.

To align with use in visual arts and our needs in annotating our graphic standards, two categories of qualities of graphical marks are represented. The class **level of visual hierarchy of graphical mark** has four subclasses. Visual hierarchy describes how prominent an element is in a composition. Subclasses were created for primary, secondary, tertiary, and quaternary levels. The class **value of graphical mark** has nine subclasses to represent qualities of marks as ordered tones (the lightness or darkness of a mark). Subclasses represent the nine-step value scale devised by Denman Ross in the early 1900s that has seven ordered tones between white and black.

#### 3.3.3. Qualities of pictures.

Describing attributes and characteristics of pictures is important for fields such as visual arts and technical communication. In the GDO, “pictures” are entities of the class pictorial graphic or pictorial sensor-generated image. The class **pictorial style** refers to whether a representation uses a diagrammatic style or a naturalistic style. **Projection type** specifies the technique employed for representing a three-dimensional referent as a two-dimensional image. **Style of representation of parts** describes the technique employed for depicting parts of the referent, such as showing a cut-away of the exterior or exploded parts [42]. **View** describes the positional relation between the referent and the line of sight of the viewer within the scene that is depicted. The anatomy extension of the GDO adds **anatomical view** and its subclasses **anatomical planar view** and **anatomical surface view**. These qualities are important for describing whether an anatomical graphic depicts a posterior view, right lateral view, or one of many other possible views. These views are not limited to human anatomy, but cover views relevant to veterinary medicine, radially symmetric organisms (such as sea urchin, which is used by developmental biologists as a model organism), and plants.

**Fig 4:**
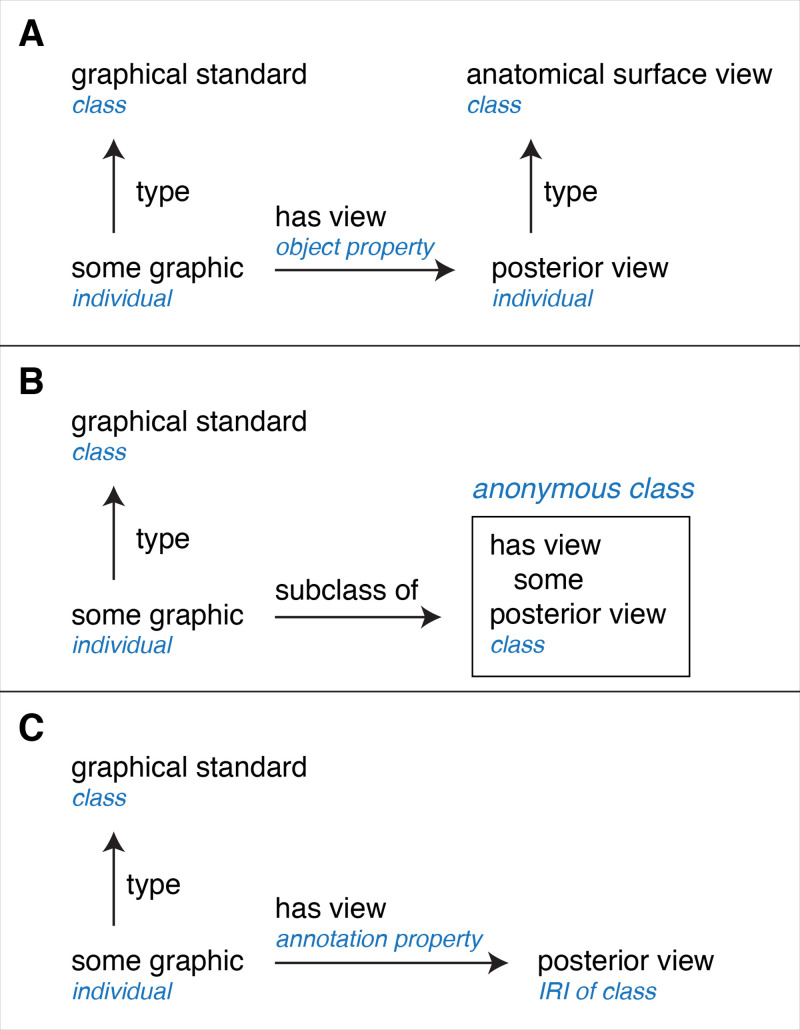
Three alternatives for representing a quality of a pictorial illustration in OWL, using the example of a graphic and posterior view.

Our purpose for developing the GDO and its anatomy extension is to create statements about graphic standards that are expressed in OWL and will support reasoning (in the broad sense of deriving meaning, not limited to description logic inference). For example, we need to state that graphic standard HUMAN0000229 shows a posterior view. Fig 4 shows three ways of modeling this information. Each graphic standard is an individual of type **graphical standard**. OWL does not allow object properties to relate individuals to classes. Options for OWL-compliant modeling are to (a) create “posterior view” as an individual and “has view” as an object property, (b) create an anonymous class of individuals that have a view of “posterior view” and relate this to the graphic using a subclass relation, or (c) create “has view” as an annotation property.

For the initial release of the GDO and anatomy extension we chose the first option, modeling the leaf nodes of the hierarchy for **pictorial quality** as individuals (option A). There are two reasons behind this decision. The first is pragmatic. We are able to state in OWL that the graphic HUMAN0000229 has a posterior view using this triplet of IRIs:

https://endlessforms.info/graphics/HUMAN0000229 (graphic of back of skull)
https://endlessforms.info/gdo/GDO_0000245 (has view)
https://endlessforms.info/gdo/GDOA_0000065 (posterior view)

The second reason for choosing to model using individuals is that there is a compelling argument for choosing to model pictorial qualities as individuals. The quality “posterior view” refers the same thing when applied to different graphics—all graphics with a posterior view are drawn as if the viewer is positioned behind the anatomical referent in an imaginary scene. Likewise, the qualities “style of cut-away of exterior” and “style of exploded parts” can reasonably be considered single entities, even if the way a style is employed in across different graphics varies somewhat.

We considered other options for this modeling before deciding on this approach. Option B of Fig 4, creating an anonymous class, was not chosen because it would complicate retrieval of the information in SPARQL by requiring increased complexity of queries. Option C, using “has view” as an annotation property, would make this information inaccessible to the logical model because annotations are treated as metadata that are ignored by most reasoners. We also decided against using punning, in which the nodes are modeled as both classes and individuals, because this adds confusion to the model.

### 3.4. Roles

**Fig 5:**
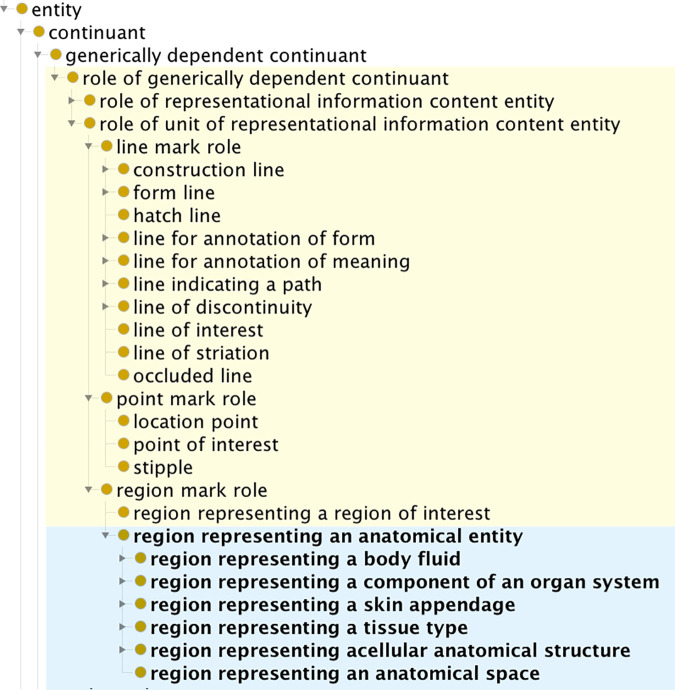
Part of the GDO/A class hierarchy for roles of generically dependent continuants. Classes created for the GDO are highlighted in yellow. Classes of the anatomy extension are in blue.

Graphical marks have roles because they serve a representational purpose when they are part of a spatial composition. A single graphical mark may have two different roles when used in two different compositions. A role in the BFO is a specifically dependent continuant that inheres in some independent continuant. As with representing qualities, representing roles presents a modeling problem because graphics are generically dependent continuants. We introduce **role of generically dependent continuant**, as shown in Fig 5, defined as “A generically dependent continuant that is assigned to or possessed by some other generically dependent continuant to bring about some result. A role does not alter the make-up of the other generically dependent continuant.”

#### 3.4.1. Roles of line marks.

**Fig 6:**
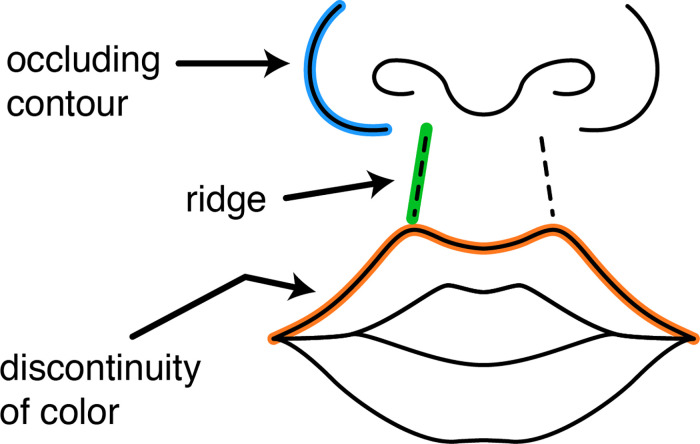
Examples of different roles of line marks in a graphic depicting the anterior view of a child’s nose and lips.

The lines of line drawings serve different representational purposes. Fig 6 demonstrates three line roles in a graphic depicting the anterior view of a child’s nose and lips. A line mark role of **occluding contour line** (also known as a “silhouette line”) is assigned to a line mark that represents the delineation of regions of surfaces visible to the viewer of the referent from regions of surfaces hidden from the viewer. A role of **ridge line** is assigned to a line mark that represents the highest points of a ridge. A role of **line of discontinuity of color** is assigned to a line mark that represents a discontinuity in the color of the referent. Individual illustrators creating this type of graphic may draw the line marks in slightly different locations or use different line weights or colors, but all would rely on the convention of using lines to represent occluding contours, ridges, and discontinuity of color.

The GDO contains an extensive hierarchy of over thirty line mark roles. Each line element of our graphic standards is assigned a Cascading Style Sheet (CSS) class in the SVG file that corresponds to the role of the line. This allows for semantic styling of the graphics by linking the visual appearance of a line to the semantic meaning of the line [39]. For example, lines with a role of ridge line appear as dashed lines when styled with the default CSS.

#### 3.4.2. Roles of region marks.

In our work designing graphical standards for anatomy, graphics are designed with bounded paths that specify regions. These regions have the role of representing some type of anatomical structure, such as a body fluid (blood, urine), a component of an organ system (vein, artery), or a portion of tissue (bone tissue, skeletal muscle tissue). The anatomy extension of the GDO has a class hierarchy of over twenty types of region mark roles for representing anatomical structures. The structure of the class hierarchy is based loosely on the class hierarchy of the Foundational Model of Anatomy (FMA) ontology for human anatomy [36]. Each leaf node of the hierarchy is mapped to the corresponding anatomical structure in the FMA and the Uberon multi-species anatomy ontology [37] using the “related anatomy” annotation property.

#### 3.4.3. Roles of representational information content entities.

We provide three classes to specify roles for different types of spatial compositions. **Pictorial representation role** is assigned to individuals of class **pictorial sensor-generated image** or **pictorial graphic**. **Data representation role** is assigned to individuals that are a **data graphic** and **relational representation role** to individuals that are a **relational graphic**. These are specified as an existential restriction. An example is:

**pictorial representation role** subClassOf (***information role of*** some (**pictorial sensor-generated image** or **pictorial graphic**))

### 3.5. Referents

**Fig 7:**
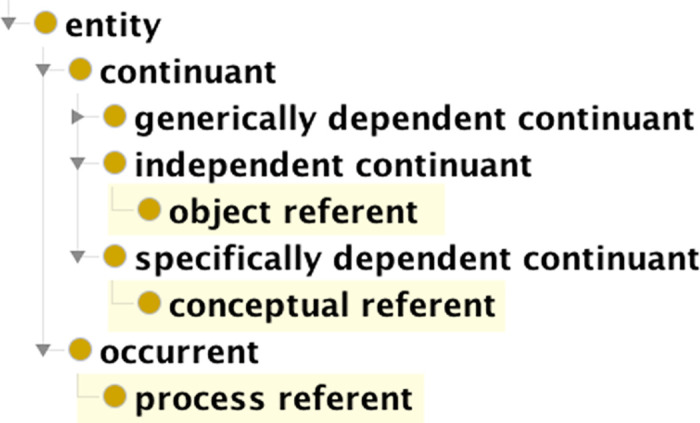
Classes for referents placed into the BFO class hierarchy.

Three classes for referents of representations were added to the GDO to align this work with theories of information, semiotics, and cognition. Placement in the class hierarchy is shown in Fig 7. An **object referent** is an independent continuant. It is typically a material entity, but may also be an immaterial entity. A referent that exists only in someone’s mind is a **conceptual referent** and classified as a specifically dependent continuant. The independent continuant in which it inheres is the person’s cognitive system [43]. A **process referent** is an occurrent.

### 3.6. Object properties

**Fig 8:**
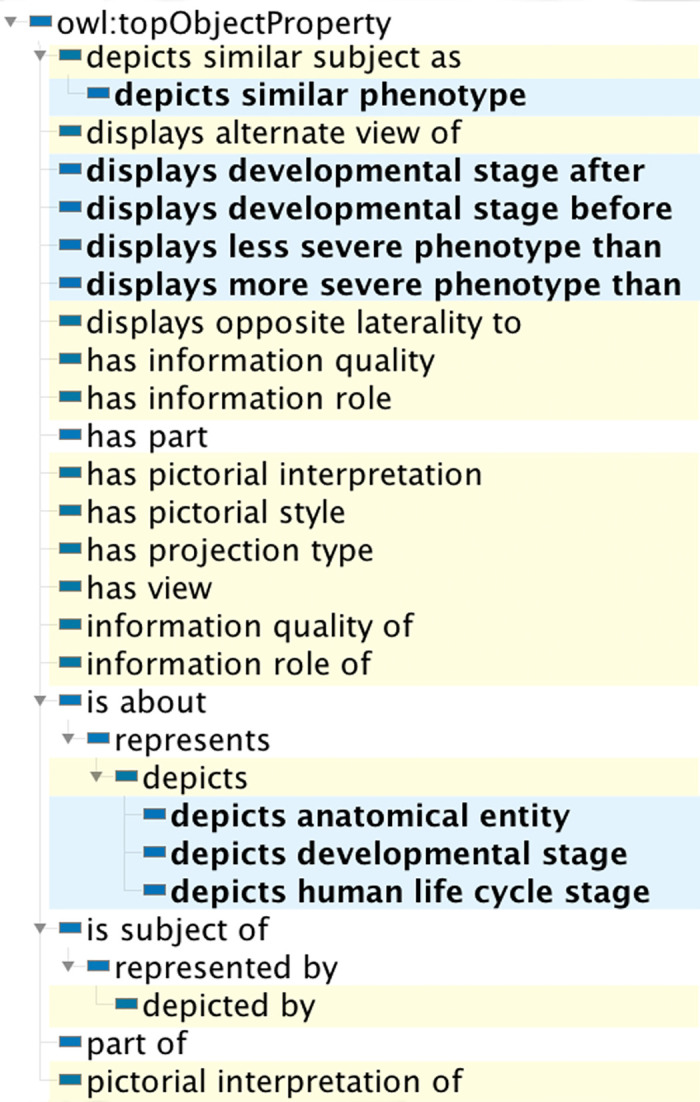
Object property hierarchy for the GDO/A. Properties created for the GDO are highlighted in yellow. Properties of the anatomy extension are in blue.

Object properties were added to the GDO/A to enable authoring of OWL-compliant statements about the anatomical information resources we create (graphic libraries and photographs of anatomical specimens). Therefore object properties were designed to apply to the broad category of pictorial spatial compositions when appropriate, as shown in Table 2. The object property hierarchy is shown in Fig 8.

**Table 2:** Examples of object properties of the GDO and their domains and ranges.

Object property	Domain	Range
*depicts similar subject as*	pictorial graphic or pictorial sensor-generated image	pictorial graphic or pictorial sensor-generated image
*depicts alternate view of*	pictorial graphic or pictorial sensor-generated image	pictorial graphic or pictorial sensor-generated image
*displays opposite laterality to*	pictorial graphic or pictorial sensor-generated image	pictorial graphic or pictorial sensor-generated image
*has view*	pictorial graphic or pictorial sensor-generated image	view
*has pictorial style*	pictorial graphic	pictorial style
*has projection type*	pictorial graphic	projection type

The property ***is about*** (cco:ont00001808) and its sub-property ***represents*** (cco:ont00001938) are provided by the IEO to make statements about information content entities. We created ***depicts*** as a more specific sub-property for stating that some pictorial spatial composition depicts some referent. It has inverse property ***is depicted by***. The properties ***has pictorial interpretation*** and ***pictorial interpretation of*** are inverse properties for constructing statements that link individuals that are a **pictorial sensor-generated image** and a **pictorial graphic** for cases in which a graphic was created for the purpose of interpreting a specific photograph or radiograph.

Anatomy-specific object properties are used in our information systems and APIs to relate anatomical graphics to one another. For example, each graphic standard for a phenotype of cleft lip is related to those that depict more severe and less severe phenotypes.

### 3.7. Data properties

**Fig 9:**
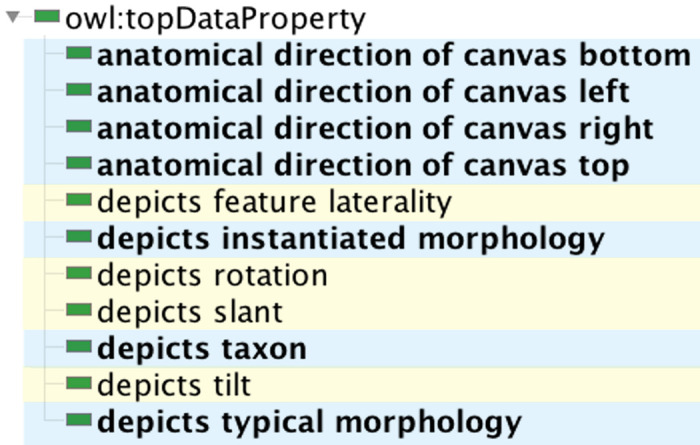
Data properties for the GDO/A. **Properties created for the GDO are highlighted in yellow.** Properties of the anatomy extension are in blue.

**Fig 10:**
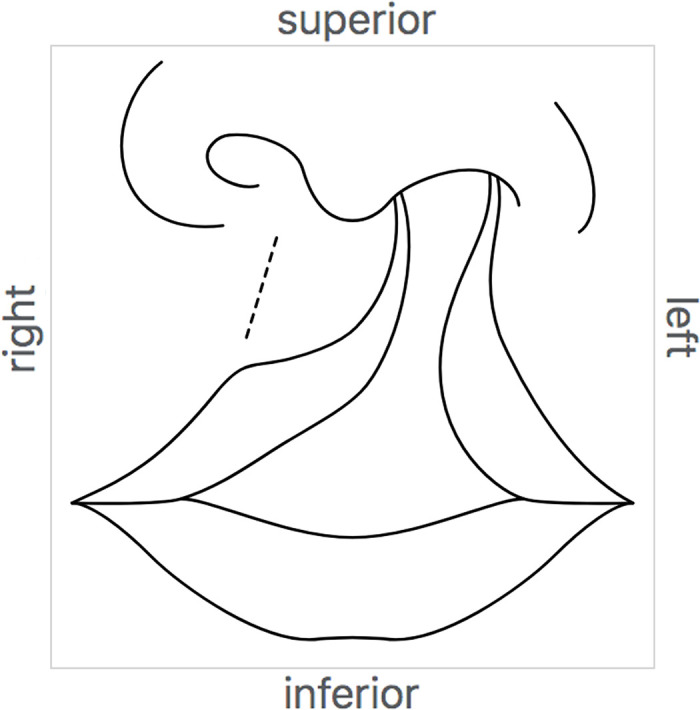
The display of graphic standard https://endlessforms.info/graphics/HUMAN0000802 on its information page. Data properties specify the anatomical directions represented by the top, bottom, left, and right of the canvas, and this information is displayed for the viewer around the edges of the canvas. Graphic reprinted under a CC BY license, with permission of Endless Forms Studio, original copyright 2024.

The GDO/A has a small number of data properties, as shown in Fig 9, to support information retrieval about our anatomical resources. Property *depicts feature laterality* has a range of {“bilateral”, “left”, “right”} and is used in the graphic library of cleft lip phenotypes to identify the laterality of the cleft. Fig 10 demonstrates use of data properties to provide information about a graphic on the webpage describing that graphic. Four properties provide information about the anatomical directions that map to the top, bottom, left, and right of the canvas a graphic is drawn on. The ranges for these properties are anatomical directions that include “right”, “dorsal”, “inferior”, and “posterior”. We chose to represent the ranges as literal values to simplify readability and query. Reusing an existing value set of anatomical directions and laterality designations would improve interoperability, however we were unable to identify such a source.

### 3.8. The GDO/A as an illustrated ontology

**Fig 11:**
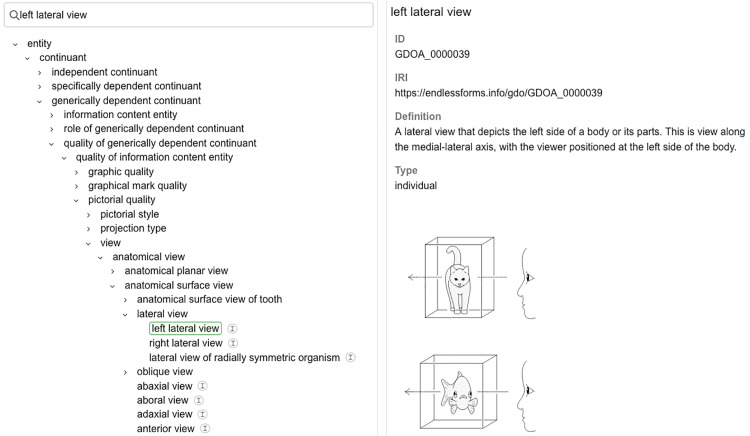
Screen capture of part of the GDO/A browser displaying class “left lateral view”. Graphics reprinted under a CC BY license, with permission of Endless Forms Studio, original copyright 2024.

To help users understand the GDO/A we have developed a web browser that presents it as an illustrated ontology. Fig 11 shows a screen capture of the browser which displays the ID, IRI, label, definition, and any associated graphics for a class. Both classes and individuals are displayed within the class hierarchy. Radio buttons allow a user to view object properties or data properties.

**Fig 12:**
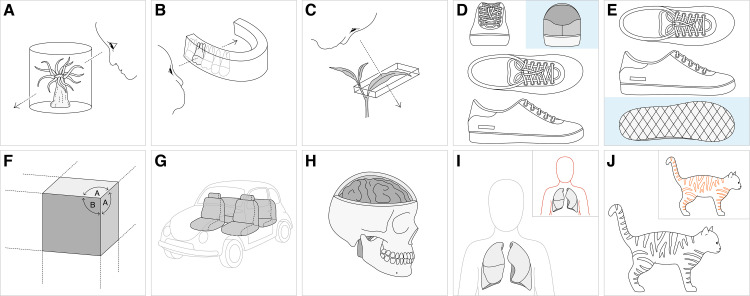
Examples of explanatory graphics developed for the GDO/A. The identifiers of the graphics form IRIs in the namespace https://endlessforms.info/graphics. **(A)** GDO0000205 illustrating “oblique superior view of radially symmetric organism”. **(B)** GDO0001068 illustrating “buccal view of tooth”. **(C)** GDO0000984 illustrating “adaxial view”. **(D)** GDO0001657 illustrating “orthographic back view”. **(E)** GDO0001670 illustrating “orthographic bottom view”. **(F)** GDO0001847 illustrating “dimetric projection”. **(G)** GDO0001326 illustrating “style of transparency of exterior”. **(H)** GDO0001332 illustrating “style of cut-away of exterior”. **(I)** GDO0001994 illustrating line role “reference form line”. **(J)** GDO0001997 illustrating line role “line of discontinuity of color”. Graphics reprinted under a CC BY license, with permission of Endless Forms Studio, original copyright 2024.

Over 100 explanatory graphics are provided to serve as visual definitions or examples. They correspond to the leaf nodes of hierarchies for **projection type**, **style of representation of parts**, **view**, and **line mark role**. Anatomical views are represented using both humans and other types of organisms as appropriate. Examples of graphics are provided in Fig 12.

### 3.9. Use of the GDO

**Fig 13:**
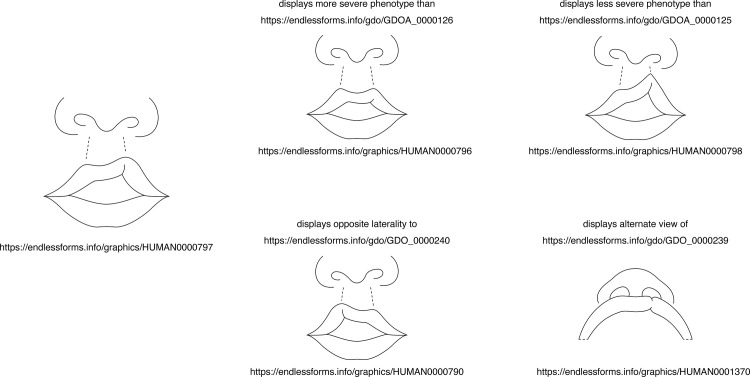
Example of using object properties to form statements relating graphics within our libraries to each other. For example, cleft lip graphic HUMAN0000797 (shown on left) displays more severe phenotype than (property GDOA_0000126) graphic HUMAN0000796. Graphics reprinted under a CC BY license, with permission of Endless Forms Studio, original copyright 2024.

The GDO is used for annotation of all graphics in our graphic libraries. As of March 2026, 3518 path elements within 445 graphics have been assigned style classes corresponding to line mark roles (see Fig 6 for examples), and 393 path elements within 260 graphics have region mark roles. Classes for anatomical views or orthographic views are associated with 340 graphics. The canvases of all anatomical graphics are associated with data properties that specify the anatomical directions represented by each of the four sides (see Fig 10). In addition, object properties are used to related graphics of phenotypes to one another, as demonstrated in Fig 13.

## 4. Discussion

This project provides a case study in developing ontological modeling for visual representations.

### 4.1. Modeling issues raised in this work

We encountered three issues that warrant further study: the first is roles and qualities, the second is the nature of information bearing entities for digital information artifacts, the third is the use of object properties for authoring OWL-compliant statements.

Issue 1: Roles and qualities. To facilitate compatibilities with other ontologies, this work uses the BFO as an upper-level ontology and extends several classes from the IEO. We encountered some difficulty in fitting our modeling for this domain into the BFO framework as we understand it. Qualities and roles in BFO modeling inhere in independent continuants. To describe qualities and roles of information content entities, which are generically dependent continuants, we created classes **quality of generically dependent continuant** and **role of generically dependent continuant**. If our modeling is consistent with the intent of BFO authors and would be useful to other ontology developers, it would be more appropriate to place these classes in BFO so they can be more easily reused by others.

Issue 2: Information bearing entities for digital information artifacts. We authored our ontology without creating classes for information bearing entities because our graphics are classified as information content entities. However, in early experiments we classified “graphics” as information bearing entities because there is something material that is perceived and people tend to call that thing a graphic. In this early modeling, graphics could be assigned roles and qualities because they were independent continuants. We abandoned modeling graphics as information bearing entities due to difficulty in accounting for differences between types of media and because it is the composition of marks or pixels that is of importance in our work. Some of the issues we encountered in representing graphics as information content entities may arise from the differences between physical information media and digital information media. Explanations provided by BFO authors to describe information bearing entities and information content entities refer to traditional media, such as a photograph or a vinyl record. In these examples it is clear that the photographic paper with silver grains or the vinyl serves as the information bearing entity. Similarly, the pattern of graphite of a pencil drawing or the paint of an oil painting is an information bearing entity. But in the case of graphics displayed on a computer monitor, what is the information bearing entity? The material of the monitor? It might be the photons of light emitted by the screen, but this would not be consistent with BFO because information bearing entities are limited to material entities.

Issue 3: Use of object properties. One purpose of this work is to create OWL-compliant statements about the graphics we create. We modeled the leaves of the class hierarchy for **pictorial quality** as individuals because OWL does not allow object properties to relate individuals to classes, only individuals to individuals. We have argued that “posterior view” can reasonably be represented as an individual. This argument may be less convincing for other types of pictorial qualities such as **pictorial style** (with individuals “diagrammatic style” and “pictorial style”) that are not as well-defined in their meaning. If the purpose of the GDO/A was solely to provide annotations for linked data, rather than serve in OWL-compliant information systems, modeling pictorial qualities as classes would add less complexity to the model.

### 4.2. Contributions of this work to the fields of visual representation and ontological modeling

This work makes a contribution to the field of ontology by modeling visual representations. As discussed in the Introduction, several other ontologies represent information artifacts but do not provide detailed modeling of visual representations. The GDO extends the Information Entity Ontology (IEO) using the classes **directive information content entity** and **representational information content entity.** In this way we distinguish between (1) information content entities that are the instructions for drawing a composition or an element of that composition (for example, **PNG file** and **SVG circle element**), and (2) information content entities that represent something (such as **data graphic**, **pictorial graphic**, and **sensor-generated image**). This modeling accommodates visual representations created in both digital and traditional media. Representational information content entities are classified based on the fundamental units from which they are created. For sensor-generated images created with a digital sensor, the fundamental unit is a **pixel of sensor-generated image** which represents a value of a measurement. For drawings made with points, lines, and regions, these fundamental units are of classes **point mark**, **line mark**, and **region mark**. When a drawing is displayed on a computer monitor and rendered using the physical pixels of the monitor it retains these fundamental units of representation. Likewise, if a photograph is enlarged on a computer monitor, the units of representation (pixels if captured by a sensor, grains of the film if captured with an analog camera) remain the same; each unit is simply rendered using an increased number of physical pixels of the monitor.

A small number of classes in the GDO are similar to classes in other ontologies. The IAO was developed to represent parts of scientific and clinical documents. It includes the class **figure** (IAO:0000308) with subclasses **image** (IAO:0000101) and **diagram** (IAO:0000309). We avoided the term “figure” in the GDO because the meaning differs across different domains of visual communication (in artistic fields, this typically referring to a drawing of a person). This IAO class is roughly equivalent to **spatial composition** in the GDO. We also avoided the term “image” in the GDO due to divergent use across fields. This IAO class is similar to the GDO class **sensor-generated image**, but our definition includes both two- and three-dimensional representations. A diagram as modeled by IAO includes both **data graphic** and **relational graphic** classes in the GDO.

The term “pixel” in the National Cancer Institute Thesaurus is defined as “The smallest resolvable rectangular area of an image, either on a screen or stored in memory.” This is similar to our class **pixel of sensor-generated image**, defined as “The fundamental unit of representation in a digital sensor-generated image. A pixel represents a value of a measurement.” The NCIT definition is more general and allows for image manipulation, while our definition is more specific because it represents a measurement.

GDO modeling is also relevant to work in image annotation. For example, the Atlas Ontology Model (AtOM) is a standard for representing brain atlases [44]. It includes “parcellation annotations” which are graphical marks that that designate boundaries or regions of brain structures represented within a brain image obtained through histological sectioning or radiographical methods.

### 4.3. Limitations of validation

At this stage of development, the GDO and the anatomy extension of the GDO have only been applied to our graphics that serve as visual standards for anatomy and the explanatory graphics accompanying the GDO/A. The GDO/A has yet to be applied to graphics from other creators or in other domains. While no formal external evaluation is currently planned, we acknowledge the need to review and expand the GDO/A when applied by other users. To capture requests or issues as they arise, we have enabled an issue tracker in the GitHub repository.

### 4.4. Envisioning use of the GDO by AI/ML-enabled applications

The GDO/A was developed to provide machine-readable information for describing our graphics of anatomy that serve as visual standards. But more broadly, we envision three types of uses for GDO/A annotations. The first is to aid machines in understanding what is depicted in a graphic. By relating regions of graphics to anatomical entities and specifying the view depicted, information systems can identify graphics that are relevant to a query. A second use is interpreting other visual representations. For example, a system that can interpret the positions and shapes of anatomical structures within a graphic can apply that information to identifying the corresponding structures in a collection of photos. Finally, annotating collections of line drawings with the representational purpose of every line offers an opportunity to train systems to understand the semantics of line drawings and to generate new drawings.

### 4.5. Access and use

In addition to our web browser at https://gdo.endlessforms.info, the GDO/A is available at https://github.com/endlessforms-info/GDO-GDOA and through the NCBO Bioportal at https://bioportal.bioontology.org/ontologies/GDOA. Requests for additions to the ontology can be submitted through the issue tracker at GitHub. Explanatory graphics are available at https://graphics.endlessforms.info. The ontology is available with a Creative Commons Attribution 4.0 License (CC BY). Graphics are provided with a Creative Commons Attribution-NonCommercial 4.0 License (CC BY-NC).
